# Epidemiology of patients with central nervous system infections, mainly neurosurgical patients: a retrospective study from 2012 to 2019 in a teaching hospital in China

**DOI:** 10.1186/s12879-021-06561-2

**Published:** 2021-08-17

**Authors:** Zheng Zhang, Yan Song, Jianbang Kang, Surong Duan, Qi Li, Fuqiang Feng, Jinju Duan

**Affiliations:** 1 Department of Pharmacy, School of Pharmacy, Shanxi Medical University, Taiyuan, Shanxi People’s Republic of China; 2 Department of Pharmacy, Second Hospital of Shanxi Medical University, No 382, Wuyi Road, Xinghualing District, Taiyuan, Shanxi People’s Republic of China; 3 Department of Information Management, Second Hospital of Shanxi Medical University, No 382, Wuyi Road, Xinghualing District, Taiyuan, Shanxi People’s Republic of China; 4 Department of Neurosurgery, Second Hospital of Shanxi Medical University, No 382, Wuyi Road, Xinghualing District, Taiyuan, Shanxi People’s Republic of China

**Keywords:** Central nervous system infections, North China, Pathogens distribution, Antibiotic susceptibility, Risk factors

## Abstract

**Background:**

Central nervous system (CNS) infections are relatively rare but are associated with high mortality worldwide. Empirical antimicrobial therapy is crucial for the survival of patients with CNS infections, and should be based on the knowledge of the pathogen distribution and antibiotic sensitivities. The aim of this study was to investigate the features of pathogens in patients with CNS infections in North China and evaluate the risk factors for mortality and multi-drug-resistant (MDR) bacterial infections.

**Methods:**

A retrospective study was conducted with patients with positive cerebrospinal fluid (CSF) cultures in a teaching hospital from January 2012 to December 2019. The following data were collected: demographic characteristics, laboratory data, causative organisms and antimicrobial sensitivity results. Data were analyzed with SPSS 16.0. Univariate analysis and binary logistic regression analyses were performed to identify the risk factors for mortality and MDR bacterial infections.

**Results:**

A total of 72 patients were diagnosed with CNS infections, and 86 isolates were identified. The proportions of Gram-positive bacteria, Gram-negative bacteria and fungi were 59.3, 30.2 and 10.5%, respectively. The predominant Gram-positive bacteria was Coagulase-negative *Staphylococci*. *Acinetobacter baumannii*, *Escherichia coli* and *Klebsiella* spp. were the predominant Gram-negative bacteria. Compared to 2012–2015 years, the proportion of Gram-negative bacteria increased markedly during 2016–2019 years. Coagulase-negative *Staphylococci*, *Streptococcus pneumoniae* and *Enterococcus faecium* had 100% sensitivity to vancomycin, teicoplanin and linezolid. *Acinetobacter baumannii* and *Klebsiella pneumoniae* were 100% sensitive to tigecycline. *Escherichia coli* had 100% sensitivity to amikacin, meropenem and imipenem. The overall mortality rate in the 72 patients was 30.6%. In multivariate analysis, age > 50 years, pulmonary infections and CSF glucose level < the normal value were associated with poor outcomes. CSF adenosine deaminase level > the normal value and the presence of external ventricular drainage/lumbar cistern drainage were associated with MDR bacterial infections.

**Conclusions:**

The mortality rate due to CNS infections reached 30.6% in our study. The proportion of Gram-negative bacteria has increased markedly in recent years. We should give particular attention to patients with risk factors for mortality and MDR bacterial infections mentioned above.

## Background

Central nervous system (CNS) infections, including meningitis, encephalitis, and brain abscesses, can occur as complications following neurosurgical operations or they can occur spontaneously [1, 2]. CNS infections cause significant mortality worldwide, resulting in a poor prognosis with a prolonged hospital stay and increased costs [1–5]. More than 100 pathogens have been reported to cause CNS infections, including bacteria, viruses, fungi and parasites [5]. The most common bacterial pathogens are *Neisseria meningitidis*, *Streptococcus pneumoniae*, Coagulase-negative *Staphylococci* (CoNS), *Staphylococcus aureus* and *Acinetobacter baumannii* [6, 7]. Cerebrospinal fluid (CSF) culture is commonly used to determine the pathogens causing CNS infections. However, the final results of culture, including antimicrobial susceptibility testing, cannot be obtained within 48 h [8, 9]. Gram staining and agglutination tests can only preliminarily distinguish among Gram-positive, Gram-negative and fungi. Thus, empirical antimicrobial therapy is essential while the pathogen identification and antimicrobial susceptibility test results are still pending, and should be based on the knowledge of the prevalence of various bacterial organisms and their antibiotic sensitivity. However, antibiotic therapy for CNS infections has been limited due to the inefficiency of drug transport across the blood–brain barrier (BBB) and the emergence of multi-drug-resistant (MDR) bacteria [6, 10–12]. In addition, the prevalence of pathogens causing bacterial infections varies based on time, geographical distribution and underlying medical conditions [13]. China has a vast territory, and the pathogen distribution differs across the country. Thus, the availability of local bacterial prevalence and antibiotic sensitivity data could help improve the empirical administration of antimicrobial therapy.

Furthermore, understanding the risk factors for mortality and MDR bacterial infections would assist clinicians in implementing interventions in a timely manner to improve patient outcomes. There were numerous studies reported the potential risk factors associated with the incidence of CNS infections in recent decades [14–16], however, data on the risk factors associated with mortality and MDR bacterial infections are limited. Herein, we present epidemiological research on CNS infections at a tertiary teaching hospital in North China and assess the crucial risk factors affecting patient outcomes and the development of MDR bacterial infections.

## Methods

### Data collection

This retrospective study was carried out at the Second Hospital of Shanxi Medical University, a 2700-bed tertiary teaching hospital in Shanxi, China. This study registered in an eight-year period (from January 2012 to December 2019). The data from patients with positive CSF cultures reported by the microbiological laboratory were obtained from the electronic medical records. For each patient, three main sets of records were collected. The first set of records was related to the general demographic characteristics, including age, sex, department, comorbidities, hospital length of stay, surgery and placement of invasive devices. The second set of records was related to the pathogen isolates and their antimicrobial susceptibility results. Duplicate isolates from a patient within 7 days were excluded. The third set of records included the following laboratory test results: the glucose level, protein level, chlorine level, adenosine deaminase (ADA) level, leukocyte count and erythrocyte count in the CSF; the white blood cell count, neutrophil count and percentage of neutrophils in the blood; the levels of liver function indicators, including alanine aminotransferase (ALT), aspartate aminotransferase (AST), total bilirubin (TBIL), alkaline phosphatase (ALP), total protein and albumin; and the levels of renal function indicators, including creatinine and urea nitrogen. We included the results of laboratory tests performed close to the time at which the CSF samples were collected.

### Microbiology and antimicrobial susceptibility testing

After the CSF samples were received, centrifugation and gram stain were performed to suggest the bacterial species initially. Blood agar plates, chocolate agar plates and MacConkey agar plates were used for bacterial culture, and plates are incubated in carbon dioxide at 35 °C. Isolates were identified by the Vitek 2 automated system (Biomerieux, France). Antimicrobial susceptibility of bacteria was tested by Kirby-Bauer disk diffusion method and interpreted according to the latest standards of Clinical and Laboratory Standards Institute (CLSI) Guidelines.

### Definitions

CNS infections was diagnosed according to the definitions of Centers for Disease Control and Prevention (CDC) as follows [17]: (1) isolation of pathogens from CSF; (2) patient was considered at least one of the following signs with no other recognized cause: fever (> 38 °C), headache, stiff neck, meningeal signs, cranial nerve signs, changing level of consciousness, or confusion; (3) increased white cells, elevated protein, and/or decreased glucose in CSF. MDR is defined as non-susceptibility to three or more classes of antibiotics, and extensively drug-resistant (XDR) is defined as non-susceptibility to at least one agent in all but two or fewer antimicrobial categories [18, 19]. The study population was divided into two groups respectively according to whether the patients survived and whether the patients detected with MDR bacteria in the CSF.

### Statistical analysis

Data were analyzed with SPSS software version 16.0. The statistical results for continuous data were recorded as mean ± SD or median (IQR) values according to the statistical distribution, and categorical parameters as number (%). To compare the difference between two groups of patients (survivors/non-survivors or MDR-positive/MDR-negative), categorical variables were analyzed by a Chi-square test or Fisher’s extract test, as required. Independent samples t-test was used for continuous variables with normal distribution, and continuous variables with non-normal distribution were compared by using the independent samples Mann–Whitney U test. Two-tailed tests were used to determine statistical significance and a *P* value < 0.05 was considered statistically significant. Factors with a *P* value < 0.1 in univariate Chi-square test or Fisher’s extract test were included in a binary logistic regression model to identify the independent risk factors. Laboratory data were converted to dichotomous variables by selecting a cut-off point based on the maximum or minimum referenced values.

## Results

### Study population

From January 2012 to December 2019, we obtained 111 positive CSF culture samples from 98 patients; 22 patients were excluded because they did not meet the diagnostic criteria for a CNS infections. In addition, 4 patients were removed because they were diagnosed with viral meningitis by the attending clinicians. Finally, 72 patients were diagnosed with CNS infections caused by bacteria or fungi, and 86 isolates were identified. Table 1 shows the characteristics of the study population.

**Table 1:** Clinical characteristics of the study population (N = 72)

Characteristics	Value
Age (years)	49.7 ± 18.3
Age > 50	41 (56.9)
Sex	
Male	54 (75)
Female	18 (25)
Department	
Neurosurgery	52 (72.2)
Neurology	15 (20.8)
Other ^a^	5 (6.9)
Days in hospital	29.5 (19.3–46.0)
Comorbidity	
Pulmonary infection	35 (48.6)
Hypertension	29 (40.3)
Hydrocephalus	26 (36.1)
Intracranial hemorrhage	19 (26.4)
Diabetes	10 (13.9)
Glucocorticoid therapy > 3 days	29 (40.3)
Surgery	
Yes	50 (69.4)
No	22 (30.6)
Invasive device	
External ventricular drainage/Lumbar cistern drainage	41 (56.9)
Catheter	57 (79.2)

^a^Rheumatology (n = 1), Cardiothoracic surgery (n = 1), Oncology (n = 1), Hematology (n = 1), Intensive Care Unit (n = 1)

### Microbiology

The distribution of the 86 causative agents is shown in Table 2. Among all the microorganisms detected, the proportions of Gram-positive bacteria, Gram-negative bacteria and fungi were 59.3, 30.2 and 10.5%, respectively. The predominant Gram-positive isolate was CoNS (43.0%), followed by *Enterococcus faecium* (5.8%) and *Streptococcus pneumoniae* (5.8%). *Acinetobacter baumannii* (7.0%), *Escherichia coli* (7.0%) and *Klebsiella* spp. (5.8%) were the common Gram-negative strains. In addition, 9 fungal isolates were identified, 6 of which were *Cryptococcus neoformans*.

**Table 2:** Pathogens isolated from patients with CNS infections

Pathogens	n (%)	2012–2015 years n (%)	2016–2019 years n (%)
Total	86	39	47
Gram-positive bacteria	51 (59.3)	30 (76.9)	21 (44.7)
Coagulase-negative *Staphylococci* ^a^	37 (43.0)	24 (61.5)	13 (27.7)
* Enterococcus faecium*	5 (5.8)	4 (10.3)	1 (2.1)
* Streptococcus pneumoniae*	5 (5.8)		5 (10.6)
* Staphylococcus aureus*	2 (2.3)	2 (5.1)	
* Enterococcus faecalis*	1 (1.2)		1 (2.1)
* Streptococcus anginosus*	1 (1.2)		1 (2.1)
Gram-negative bacteria	26 (30.2)	6 (15.4)	20 (42.6)
* Acinetobacter baumannii*	6 (7.0)	1 (2.6)	5 (10.6)
* Escherichia coli*	6 (7.0)	3 (7.7)	3 (6.4)
* Klebsiella* spp. ^b^	5 (5.8)		5 (10.6)
* Enterobacter cloacae*	2 (2.3)		2 (4.3)
* Enterobacter aerogenes*	2 (2.3)		2(4.3)
* Pseudomonas aeruginosa*	1 (1.2)		1 (2.1)
* Acinetobacter lwoffii*	1 (1.2)		1 (2.1)
* Stenotrophomonas maltophilia*	1 (1.2)	1 (2.6)	
* Pseudomonas oryzihabitans*	1 (1.2)		1 (2.1)
* Brucella* spp.	1 (1.2)	1 (2.6)	
Fungus	9 (10.5)	3 (7.7)	6 (12.8)
* Cryptococcus neoformans*	6 (7.0)	2 (5.1)	4 (8.5)
* Aspergillus fumigatus*	1 (1.2)		1 (2.1)
* Candida parapsilosis*	1 (1.2)	1 (2.6)	
* Candida tropicalis*	1 (1.2)		1 (2.1)

^a^*Staphylococcus haemolyticus* (n = 11), *Staphylococcus epidermidis* (n = 4), *Staphylococcus hominis* (n = 1), unidentified Coagulase-negative *Staphylococci* (n = 21)

^b^*Klebsiella pneumoniae* (n = 3), *Klebsiella oxytoca* (n = 2)

**Fig. 1:**
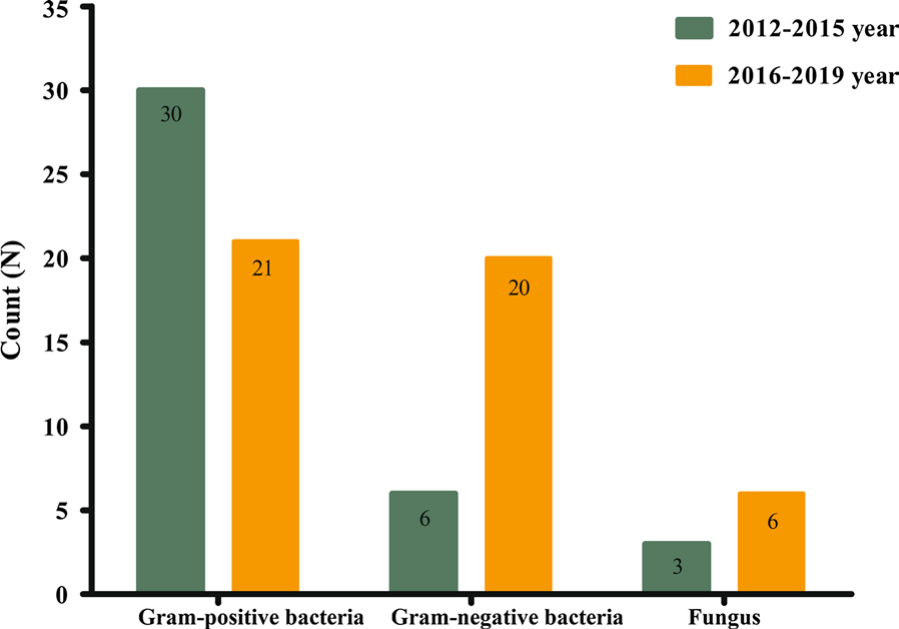
The pathogens distribution in different periods

The changes in pathogen distribution across different periods are shown in Fig. 1 and Table 2. We observed that Gram-positive bacteria were still the dominant pathogens, but the detection rate of Gram-negative bacteria increased markedly. The proportion of Gram-negative bacteria relative to the total number of pathogens was 15.4% in the period from 2012 to 2015 and 42.6% in the period from 2016 to 2019. Moreover, fungi were also found more frequently in the later period. In particular, *Streptococcus pneumoniae* and *Klebsiella* spp. were identified only in the period from 2016 to 2019. Furthermore, we compared the pathogen distributions between the neurosurgery and non-neurosurgery groups (Table 3).

**Table 3:** Pathogens distribution of neurosurgery and non-neurosurgery groups

Pathogens	n (%)	Neurosurgery n (%)	Non-neurosurgery^1^ n (%)
Total	86	64	22
Gram-positive bacteria	51 (59.3)	43 (67.2)	8 (36.4)
Coagulase-negative *Staphylococci*^a^	37 (43.0)	35 (54.7)	2 (9.1)
* Enterococcus faecium*	5 (5.8)	5 (7.8)	
* Streptococcus pneumoniae*	5 (5.8)	1 (1.6)	4 (18.2)
* Staphylococcus aureus*	2 (2.3)	1 (1.6)	1 (4.5)
* Enterococcus faecalis*	1 (1.2)		1 (4.5)
* Streptococcus anginosus*	1 (1.2)	1 (1.6)	
Gram-negative bacteria	26 (30.2)	19 (29.7)	7 (31.8)
* Acinetobacter baumannii*	6 (7.0)	6 (9.4)	
* Escherichia coli*	6 (7.0)	4 (6.3)	2 (9.1)
* Klebsiella* spp.^b^	5 (5.8)	3 (4.7)	2 (9.1)
* Enterobacter cloacae*	2 (2.3)	1 (1.6)	1 (4.5)
* Enterobacter aerogenes*	2 (2.3)	2 (3.1)	
* Pseudomonas aeruginosa*	1 (1.2)	1 (1.6)	
* Acinetobacter lwoffii*	1 (1.2)		1 (4.5)
* Stenotrophomonas maltophilia*	1 (1.2)	1 (1.6)	
* Pseudomonas oryzihabitans*	1 (1.2)	1 (1.6)	
* Brucella* spp.	1 (1.2)		1 (4.5)
Fungus	9 (10.5)	2 (3.1)	7 (31.8)
* Cryptococcus neoformans*	6 (7.0)		6 (27.3)
* Aspergillus fumigatus*	1 (1.2)		1 (4.5)
* Candida parapsilosis*	1 (1.2)	1 (1.6)	
* Candida tropicalis*	1 (1.2)	1 (1.6)	

^a^*Staphylococcus haemolyticus* (n = 11), *Staphylococcus epidermidis* (n = 4), *Staphylococcus hominis* (n = 1), unidentified Coagulase-negative *Staphylococci* (n = 21)

^b^*Klebsiella pneumoniae* (n = 3), *Klebsiella oxytoca* (n = 2)

^1^Department of Neurology, Rheumatology, Cardiothoracic surgery, Oncology, Hematology, Intensive Care Unit

### Antimicrobial susceptibility testing

The in vitro antibiotic sensitivities of the Gram-positive isolates are shown in Table 4. CoNS had 80% sensitivity to rifampicin and 100% sensitivity to vancomycin, teicoplanin and linezolid. All isolates of *Streptococcus pneumoniae* were sensitive to levofloxacin, moxifloxacin vancomycin and teicoplanin. For the *Enterococcus faecium*, vancomycin, teicoplanin and linezolid were 100% sensitive antibiotics. Of the Gram-negative isolates, *Acinetobacter baumannii* were 83.3% sensitive to minocycline, and no isolates were resistant to tigecycline. *Escherichia coli* had 100% sensitivity to amikacin, meropenem and imipenem. For *Klebsiella pneumoniae*, the only antibiotic to which there was no resistance was tigecycline. The details of the antibiotic sensitivity of the Gram-negative isolates are shown in Table 5. For the fungal isolates, *Cryptococcus neoformans* had 100% sensitivity to 5-fluorouracil, amphotericin B, fluconazole and voriconazole.

**Table 4:** Antimicrobial susceptibility of Gram-positive isolates

	CoNS (N = 37)				*Streptococcus pneumoniae* (N = 5)				*Enterococcus faecium* (N = 5)			
	n	S	I	R	n	S	I	R	n	S	I	R
Penicillin	37	1 (2.7%)		36 (97.3%)	5	2 (40%)		3 (60%)	5			5 (100%)
Oxacillin	37	7 (18.9%)		30 (81.1%)	–				–			
Ampicillin	–				–				5			5 (100%)
Amoxicillin-Clavulanate	34	7 (20.6%)		27 (79.4%)	–				–			
Cefazolin	28	4 (14.3%)		24 (85.7%)	–				–			
Ciprofloxacin	16	5 (31.3%)		11 (68.8%)	–				5			5 (100%)
Levofloxacin	20	8 (40%)	5 (25%)	7 (35%)	5	5 (100%)			–			
Moxifloxacin	6	3 (50%)	1(16.7%)	2(33.3%)	5	5 (100%)			–			
Tetracycline	36	27 (75%)		9 (25%)	5		1 (20%)	4 (80%)	5	3 (60%)		2 (40%)
Erythrocin	37	4 (10.8%)		33 (89.2%)	5		1 (20%)	4 (80%)	–			
Clindamycin	37	14 (37.8%)	7 (18.9%)	16 (43.2%)	3			3 (100%)	–			
Chloramphenicol	–				5	4 (80%)		1 (20%)	5	3 (60%)	2 (40%)	
Rifampicin	35	28 (80%)		7 (20%)	2	2 (100%)			5	1 (20%)		4 (80%)
Compound Sulfamethoxazole	37	9 (24.3%)		28 (75.7%)	5	2 (40%)	1 (20%)	2 (40%)	–			
Gentamicin	37	21 (56.8%)	1 (2.7%)	15 (40.5%)	–				5	1 (20%)		4 (80%)
Vancomycin	37	37 (100%)			5	5 (100%)			5	5 (100%)		
Teicoplanin	37	37 (100%)			5	5 (100%)			5	5 (100%)		
Linezolid	37	37 (100%)			2	2 (100%)			5	5 (100%)		

*S* sensitive, *I* intermediary, *R* resistant, (–) not done

**Table 5:** Antimicrobial susceptibility of Gram-negative isolates

	*Acinetobacter baumannii* (N = 6)				*Escherichia coli* (N = 6)				*Klebsiella pneumoniae* (N = 3)				*Klebsiella oxytoca* (N = 2)			
	n	S	I	R	n	S	I	R	n	S	I	R	n	S	I	R
Piperacillin	6			6 (100%)	–				–				–			
Piperacillin Tazobactam	6			6 (100%)	6	5 (83.3%)	1 (16.7%)		3			3 (100%)	2	1 (50%)		1 (50%)
Cefazolin	–				6	1 (16.7)		5 (83.3%)	3			3 (100%)	2			2 (100%)
Cefuroxime	–				6	1 (16.7%)		5 (83.3%)	3			3 (100%)	2	1 (50%)		1 (50%)
Ceftriaxone	–				5	2 (40%)		3 (60%)	3			3 (100%)	2			2 (100%)
Ceftazidime	6	1 (16.7%)		5 (83.3%)	6	4 (66.7%)		2 (33.3%)	3			3 (100%)	2	1 (50%)		1 (50%)
Cefepime	6			6 (100%)	6	3 (50%)		3 (50%)	3			3 (100%)	2		1 (50%)	1 (50%)
Cefoxitin	–				6	5 (83.3%)	1 (16.7%)		3			3 (100%)	2	1 (50%)		1 (50%)
Cefoperazone Sulbactam	6			6 (100%)	6	4 (66.7%)	1 (16.7%)	1 (16.7%)	3			3 (100%)	2		1 (50%)	1 (50%)
Aztreonam	–				5	1 (20%)	1 (20%)	3 (60%)	2			2 (100%)	1			1 (100%)
Amikacin	6	1 (16.7%)		5 (83.3%)	6	6 (100%)			3	1 (33.3%)		2 (66.7%)	2	1 (50%)		1 (50%)
Tobramycin	6	1 (16.7%)		5 (83.3%)	–				–				–			
Ciprofloxacin	6	1 (16.7%)		5 (83.3%)	5			5 (100%)	2			2 (100%)	1		1 (100%)	
Levofloxacin	6	1 (16.7%)		5 (83.3%)	6	1 (16.7%)	1 (16.7%)	4 (66.7%)	3			3 (100%)	2		1 (50%)	1 (50%)
Compound Sulfamethoxazole	6		2 (33.3%)	4 (66.7%)	–				–				–			
Meropenem	6			6 (100%)	6	6 (100%)			3			3 (100%)	2	1 (50%)		1 (50%)
Imipenem	6			6 (100%)	6	6 (100%)			3			3 (100%)	2	1 (50%)		1 (50%)
Minocycline	6	5 (83.3%)		1 (16.7%)	–				–				–			
Tigecycline	2	2 (100%)			–				2	2 (100%)			–			

*S* sensitive, *I* intermediary, *R* resistant, (–) not done

### Risk factors for mortality

The demographic and clinical characteristics of both survivors and non-survivors with CNS infections are shown in Table 6. Of the study population, 50 patients survived, and 22 patients died, yielding a mortality rate of 30.6%. In univariate analysis, we found significant differences between the two groups in the following aspects (P < 0.05): age, pulmonary infection, hypertension, the CSF glucose level, the CSF protein level, the CSF leukocyte count, the blood percentage of neutrophils, the ALT level, the AST level, the serum albumin level, and the urea nitrogen level. In binary logistic regression analysis, we found that age > 50 years, pulmonary infection, and a CSF glucose level < the normal value were independent risk factors for mortality (Table7).

**Table 6:** Comparison of demographic and clinical characteristics between survivors and non-survivors

Characteristics	Total (N = 72)	Survivors (N = 50)	Non-Survivors (N = 22)	P
Age (years)	49.7 ± 18.3	45.5 ± 17.8	59.0 ± 16.3	0.003
Age > 50	41	23	18	0.005
Sex				
Male	54 (75.0)	36 (72.0)	18 (81.8)	0.375
Female	18 (25.0)	14 (28.0)	4 (18.2)	
Days in hospital	29.5 (19.3–46.0)	29.0 (20.7–45.0)	33.5 (18.0–51.0)	0.633
Comorbidity				
Pulmonary infection	35 (48.6)	15 (30.0)	20 (90.9)	< 0.001
Hypertension	29 (40.3)	16 (32.0)	13 (59.1)	0.031
Hydrocephalus	26 (36.1)	16 (32.0)	10 (45.5)	0.274
Intracranial hemorrhage	19 (26.4)	13 (26.0)	6 (27.3)	0.91
Diabetes	10 (13.9)	5 (10.0)	5 (22.7)	0.285
MDR bacteria infection	49 (68.1)	31 (62.0)	18 (81.8)	0.097
Glucocorticoid therapy > 3 days	29 (40.3)	18 (36.0)	11 (50.0)	0.265
Surgery				
Yes	50 (69.4)	33 (66.0)	17 (77.3)	0.339
No	22 (30.6)	17 (34.0)	5 (22.7)	
Invasive device				
External ventricular drainage/Lumbar cistern drainage	41 (56.9)	25 (50.0)	16 (72.7)	0.073
Catheter	57 (79.2)	37 (74.0)	20 (90.9)	0.189
CSF data				
ADA level (U/L)	15.50 (4.33–30.95)	14.00 (3.30–28.25)	19.55 (5.48–37.48)	0.131
Glucose level (mmol/L)	1.71 (0.21–2.98)	2.29 (1.35–3.46)	0.35 (0.05–0.91)	< 0.001
Chlorine level (mmol/L)	116.68 ± 9.27	117.16 ± 8.67	115.58 ± 10.63	0.508
Protein level (g/L)	2.37 (1.12–4.01)	1.67 (0.84–2.78)	4.00 (2.98–4.54)	< 0.001
Leukocyte count (× 10^6^/L)	340.0 (52.5–2400.0)	188.0 (27.5–927.5)	1125.0 (102.5–4185.0)	0.029
Erythrocyte count (× 10^6^/L)	395.0 (10.0–4787.5)	395.0 (7.5–1987.5)	810.0 (65.0–10,675.0)	0.306
Blood data				
White blood cell level (× 10^9^/L)	13.13 ± 6.11	12.42 ± 5.64	14.75 ± 6.94	0.138
Neutrophils level (× 10^9^/L)	10.55 (6.94–13.48)	10.13 (6.58–12.68)	12.56 (8.68–14.85)	0.071
Percentage of neutrophils	85.21 (78.86–88.05)	84.35 (76.36–87.49)	87.14 (83.40–88.88)	0.039
Liver function				
ALT (U/L)	22.80 (14.63–57.93)	20.80 (14.08–46.35)	36.60 (19.40–125.15)	0.018
AST (U/L)	22.60 (17.03–39.45)	20.05 (15.53–38.03)	31.80(19.25–66.28)	0.014
TBIL (mmol/L)	14.65 (10.13–18.60)	13.90 (9.40–18.30)	15.05 (12.88–21.48)	0.167
ALP (U/L)	89.5 (72.0–127.0)	82.0 (70.5–118.0)	104.0 (77.8–170.5)	0.069
Total protein (g/L)	63.22 ± 8.06	64.30 ± 8.04	60.76 ± 7.73	0.086
Albumin (g/L)	35.93 ± 6.62	37.75 ± 5.51	31.79 ± 7.15	< 0.001
Renal function				
Urea nitrogen (mmol/L)	5.05 (3.58–6.95)	4.72 (2.98–6.58)	6.13 (4.63–8.60)	0.02
Creatinine (mmol/L)	61.53 ± 25.25	61.27 ± 27.27	62.14 ± 20.51	0.893

**Table 7:** Risk factors for mortality

Factor	OR	95% CI	P
Age > 50 years	4.73	0.86–25.94	0.074
Pulmonary infection	38.4	6.11–241.49	< 0.001
CSF glucose level < the normal value	20.41	3.11–134.07	0.002

*OR* odds ratio, *CI* confidence interval, the normal value of CSF glucose level was 2.2–3.8 mmol/L

### Risk factors for MDR bacterial infections

Compared with MDR bacteria-negative patients, MDR bacteria-positive patients more frequently underwent surgery, had external ventricular drainage/lumbar cistern drainage, were complicated with intracranial haemorrhage, and had elevated levels of CSF ADA, protein and leukocytes (Table 8). According to the logistic regression model, a CSF ADA level > the normal value and the presence of external ventricular drainage/lumbar cistern drainage were associated with MDR bacterial infections (Table 9).

**Table 8:** Differences between MDR-positive and MDR-negative patients

Characteristics	Total (N = 72)	MDR-positive (N = 49)	MDR-negative (N = 23)	P
Age (years)	49.7 ± 18.3	50.8 ± 19.1	47.3 ± 16.6	0.459
Sex				
Male	54 (75.0)	35 (71.4)	19 (82.6)	0.307
Female	18 (25.0)	14 (28.6)	4 (17.4)	
Days from hospitalization to detection of bacteria	9.5 (3.0–17.8)	10.0 (4.5–17.5)	5.0 (1.0–18.0)	0.258
Comorbidity				
Pulmonary infection	35 (48.6)	27 (55.1)	8 (34.8)	0.108
Hypertension	29 (40.3)	23 (46.9)	6 (26.1)	0.093
Hydrocephalus	26 (36.1)	20 (40.8)	6 (26.1)	0.225
Intracranial hemorrhage	19 (26.4)	18 (36.7)	1 (4.3)	0.004
Diabetes	10 (13.9)	8 (16.3)	2 (8.7)	0.612
Surgery				
Yes	50 (69.4)	39 (79.6)	11 (47.8)	0.006
No	22 (30.6)	10 (20.4)	12 (52.2)	
Invasive device				
External ventricular drainage/Lumbar cistern drainage	41 (56.9)	34 (69.4)	7 (30.4)	0.002
Catheter	57 (79.2)	42 (85.7)	15 (65.2)	0.092
CSF data				
ADA level (U/L)	15.50 (4.33–30.95)	23.00 (5.95–36.35)	6.00 (1.50–17.10)	0.001
Glucose level (mmol/L)	1.71 (0.21–2.98)	1.65 (0.17–2.66)	2.11 (0.23–3.40)	0.476
Chlorine level (mmol/L)	116.68 ± 9.27	116.03 ± 9.5	118.04 ± 8.88	0.395
Protein level (g/L)	2.37 (1.12–4.01)	3.06 (1.65–4.11)	1.06 (0.67–2.57)	< 0.001
Leukocyte count (× 10^6^/L)	340.0 (52.5–2400.0)	480.0 (120.0–2400.0)	60.0 (8.0–2420.0)	0.033
Erythrocyte count (× 10^6^/L)	395.0 (10.0–4787.5)	800.0 (70.0–6675.0)	25.0 (0.0–1560.0)	0.052
Blood data				
White blood cell level (× 10^9^/L)	13.13 ± 6.11	13.3 ± 5.9	12.9 ± 6.7	0.798
Neutrophils level (× 10^9^/L)	10.55 (6.94–13.48)	11.20 (7.84–13.14)	9.48 (6.21–13.59)	0.48
Percentage of neutrophils	85.21 (78.86–88.05)	85.50 (80.01–87.82)	83.5 (75.9–90.3)	0.503

**Table 9:** Risk factors for MDR bacterial infections

Factor	OR	95% CI	P
CSF ADA level > the normal value	5.63	1.61–19.72	0.007
External ventricular drainage/Lumbar cistern drainage	6.02	1.85–19.64	0.003

The normal value of CSF ADA level was 0–5 U/L

## Discussion

The microbiological examination of CSF specimens serves as an important basis for the diagnosis and treatment of CNS infections. We retrospectively collected the data from patients with positive CSF culture results and analyzed the pathogen distribution, antibiotic sensitivity and risk factors for mortality. Furthermore, risk factors for MDR bacterial infections were assessed. In our study, 111 positive CSF culture specimens were identified. According to the results of retrospective analysis, 26 samples from 26 patients were considered to be false positives in our 8-year study. The occurrence of false positive culture result could be due to a lack of standardization of the available assays and uncertainty about sample collection and processing. In addition, the CSF samples could have been contaminated by normal microbial species on the skin, such as CoNS. Wong et al. and Chang et al. reported proportions of false-positive CSF culture results of 58.7% (71/121) and 11.1% (12/108), respectively [12, 20]. Thus, it is important to exclude false-positive results.

Of the 86 isolates detected in patients with confirmed CNS infections, the proportions of Gram-positive bacteria, Gram-negative bacteria and fungi in our study were 59.3, 30.2 and 10.5%, respectively. This was approximately the same as the results of previous studies [12, 21]. In our study, the top seven pathogens were CoNS (43.0%), *Acinetobacter baumannii* (7.0%), *Escherichia coli* (7.0%), *Cryptococcus neoformans* (7.0%), *Enterococcus faecium* (5.8%), *Streptococcus pneumoniae* (5.8%) and *Klebsiella* spp. (5.8%). These results did not differ significantly from those of the multi-center study of positive CSF isolates from the China Antimicrobial Surveillance Network (CHINET, www.chinets.com), as they showed that CoNS was the most common isolate (> 44.97%), followed by *Acinetobacter baumannii* (12.43%), *Klebsiella pneumoniae* (8.23%), *Enterococcus faecium* (3.95%), *Escherichia coli* (3.92%), *Staphylococcus aureus* (2.61%) and *Enterococcus faecalis* (2.42%) in 2019. Whereas, *Cryptococcus neoformans* accounted for a higher percentage in our study. Our data indicated that the age range of the 6 patients with *Cryptococcus neoformans* CNS infections was between 43 and 68 years, of whom 1 patient had tumour, two patients had hypoproteinemia and four patients had electrolyte disturbances.

However, according to the published data, the most common pathogens causing CNS infections are *Neisseria meningitidis*, *Streptococcus pneumoniae* and *Haemophilus influenzae* [22, 23]. Our results were significantly different. There are several possible reasons: (1) *Neisseria meningitidis*, *Streptococcus pneumoniae* and *Haemophilus influenzae* are fastidious bacteria, and they are difficult to grow under normal culture conditions. (2) *Neisseria meningitidis*, *Streptococcus pneumoniae* and *Haemophilus influenzae* are sensitive to most antimicrobial agents, and initial antimicrobial therapy may have contributed to the low positive culture rate. (3) In our study, 52 out of 72 patients were from neurosurgery and the majority of patients (56.9%) had undergone external ventricular drainage/lumbar cistern drainage. For patients with neurosurgery-related CNS infections, most studies have identified *Staphylococcus* spp. as the most common pathogens, particularly CoNS [12, 14], which was consistent with our results.

Therefore, we divided the patients into two groups by department. The results showed that in the neurosurgery group, the main pathogens were CoNS (54.7%), *Acinetobacter baumannii* (9.4%) and *Enterococcus faecium* (7.8%), while in the non-neurosurgery group, *Cryptococcus neoformans* (27.3%) and *Streptococcus pneumoniae* (18.2%) were the predominant pathogens. Recognizing the differences in pathogen distribution in different departments could be inform the choice of corresponding empirical treatment.

With respect to the pathogen distribution in different periods, we found that the proportion of Gram-negative bacteria increased markedly from 15.4% in the period from 2012 to 2015 to 43.5% in the period from 2016 to 2019, particularly, *Acinetobacter baumannii* from 2.6% to 10.6% and *Klebsiella* spp. from 0 to 10.6%. In recent decades, CNS infections caused by Gram-negative bacteria have attracted more attention from clinicians, especially MDR/XDR A*cinetobacter baumannii* and *Enterobacteriaceae* [24–28]. Of the Gram-positive bacteria, CoNS was still the predominant pathogen, although the percentage decreased dramatically from 61.5 to 27.7%. In addition, compared to the period from 2012 to 2015, the number of species of isolated strains increased during the period from 2016 to 2019, and *Streptococcus pneumoniae* and *Klebsiella* spp. were identified only in the later period. The cultivation of *Streptococcus pneumoniae* is challenging because autolysis results in decreased viability [28]. Advances in automated continuously monitored CSF culture systems could process positive bottles before autolysis occurs. Therefore, the increase in isolated species could be associated with the improvements in laboratory and microbiological inspection techniques.

According to the results of antibiotic sensitivity testing in this study, 81.1% (30/37) of the CoNS isolates were methicillin-resistant strains. This proportion was higher than those reported in some studies, such as 75% and approximately 55%-75% [12, 29]. Additionally, CoNS had 80% sensitivity to rifampicin. In China, rifampicin, which is usually used against tuberculosis bacilli, is rarely used to treat infections caused by other bacteria, except for severe MDR bacterial infections. *Staphylococcus* spp. had 100% sensitivity to vancomycin, teicoplanin and linezolid in the present study. Sixty percent (3/5) of the *Streptococcus pneumoniae* strains were resistant to penicillin in our study, while 5 *Streptococcus pneumoniae* strains were completely resistant to penicillin in an Ethiopian study [30]. The identified high level of resistance to penicillin, which is mainly used as a standard regimen for the empiric treatment of CNS infections caused by *Streptococcus pneumoniae*, could create difficulties with regard to clinical treatment. Moreover, several studies have reported the emergence of vancomycin-resistant *Enterococcus faecium* [31–33], yet, no vancomycin-resistant strains were identified in the present study.

83.3% (5/6) of the isolates of *Acinetobacter baumannii* were XDR bacteria in our study, while an Indian study reported that 20.8% of *Acinetobacter* isolates were XDR strains [34]. An obvious difference was the sensitivity to cefoperazone-sulbactam, approximately 74% of the *Acinetobacter* isolates were found to be sensitive to cefoperazone-sulbactam in India [34], yet the 6 *Acinetobacter baumannii* isolates were all resistant in our study. The data from two Chinese hospitals indicated that *Acinetobacter baumannii* had low sensitivity to cefoperazone-sulbactam (5.3% and 16.3%) [21, 26]. This might indicate that regional and medical conditions have substantial effects on antibiotic resistance. However, high rates of resistance to carbapenems were found in our study and other reports [21, 26, 34].

In the meantime, meningitis caused by *Enterobacteriaceae*, particularly carbapenem-resistant *Enterobacteriaceae* (CRE), remains a therapeutic challenge worldwide [27]. Among the *Enterobacteriaceae* strains in our study, carbapenem-resistant species appeared only in *Klebsiella* spp. Four of five *Klebsiella* isolates showed resistance to carbapenems, of which three *Klebsiella pneumoniae* strains were all resistant to carbapenems. In our previous study, the mechanisms of resistance in carbapenem-resistant *Klebsiella pneumoniae* strains isolated from the CSF of patients in our hospital involved New Delhi metallo-β-lactamase and *Klebsiella pneumoniae* carbapenemases. KL47 and KL22KL37 were the serotypes and ST11 was the prevalent sequence type [35]. With regard to the *Escherichia coli*, our findings showed that they had low sensitivity to cephalosporins, but they had 100% sensitivity to carbapenem antibiotics and were highly sensitive to enzyme inhibitor compound preparations. These findings might demonstrate that the major mechanism of resistance in *Escherichia coli* in our hospital is the production of extended-spectrum beta-lactamases (ESBLs) rather than carbapenemases.

Antimicrobial agents have been used successfully to treat infectious diseases for a long time. Unfortunately, the misuse and overuse of antibiotics has led to increased antibiotic resistance [36]. Antimicrobial therapy for CNS infections has been complicated by the emergence of MDR strains. With regard to Gram-negative bacteria, tigecycline and polymyxin/colistin are usually used for the treatment of MDR/XDR *Acinetobacter baumannii* and *Klebsiella pneumoniae* infections [27, 37]. Nevertheless, due to the existence of the BBB, CNS infections does not show any improvement when treated with them by intravenous administration. Hence, the intrathecal (ITH) or intraventricular (IVT) administration of these two agents has been performed in recent years [38–41]. However, ITH and IVT antibiotic therapy has not been standardized [2]. Moreover, there are no guidelines for the ITH/IVT administration of tigecycline. We need to further explore the ITH/IVT administration of antimicrobial therapy to improve the outcomes in patients with CNS infections.

A previous study showed that mortality due to bacterial meningitis ranges from 10 to 20% in high-resource settings and can be as high as 50% in lower-resource settings [8]. This study showed that the mortality rate due to CNS infections was 30.6% in our hospital. Therefore, it is important to determine the risk factors affecting the outcomes of patients with CNS infections, particularly in developing countries. Previous studies predicted survival following CNS infections and suggested that age > 40 years, the presence of external ventricular drainage, low CSF glucose levels, high CSF protein levels, a CSF leukocyte count > 200 cells/mm^3^, ICU admission, and the presence of comorbidities were risk factors for mortality [34, 42, 43]. In our analysis, age > 50 years, complicated with pulmonary infection and a CSF glucose level < the normal value were independent risk factors for mortality. Older patients are usually characterized by markedly altered organ and physiological functions that often require tailored treatment, such as individual drug administration and extracorporeal therapies. Thus, a comprehensive assessment of patient status is needed before treatment, especially for older patients. Complicated with a pulmonary infection might increase the difficulty of antibiotic therapy and lead to a poor prognosis. Low glucose level in the CSF is a crucial diagnostic criterion for CNS infections. Our findings showed that the CSF glucose level is not only an important diagnostic indicator, but also associated with a poor prognosis.

Lewin et al. reported that immunosuppressive therapy (glucocorticoids) may reduce meningeal inflammation but can also further diminish the distribution of antimicrobials throughout the CNS [10]. This could result in failure to achieve effective antimicrobial concentrations in the CNS. Twenty-nine patients were administered glucocorticoids in our study, and we analyzed the relationship between glucocorticoid therapy and patient outcomes in univariate analysis. Although the results indicated that there was not a significant association in our study, compared to the survivors, the non-survivors were more likely to have received glucocorticoid therapy (50% vs 36%). Moreover, we found that the proportion of male (75%) was significantly higher than that of female (25%). The possible explanation could be that men are more likely to smoke or suffer craniocerebral trauma. Smoking is a risk factor for cerebrovascular disease [44]. Thus, men may account for a higher proportion of patients undergoing neurosurgery, and they are also more likely to develop CNS infections. However, we did not find a significant association between sex and patient outcome.

Exploring the risk factors for mortality and MDR bacterial infections could help clinicians initiate interventions in a timely manner to improve patient outcomes. Based on our results, with regard to the management of patients with CNS infection, there are several points should be considered. First, the initial empirical treatment should be based on the distributions of pathogens in different departments, and appropriate antibiotics that can be effectively transported across the BBB should be selected. Second, clinicians should give particular attention to patients with the independent risk factors for mortality and MDR bacterial infections mentioned above. For patients with pulmonary infections, the antibiotics selected should be useful for the treatment of both the pulmonary and CNS infections, and combination therapy should be strongly considered. Patients with external ventricular drainage/lumbar cistern drainage are more likely to be infected by MDR bacteria, so it is crucial to keep the drainage tube unobstructed, provide adequate nursing care, and remove the drainage tube as early as possible. Meanwhile, nosocomial infection control measures should be strengthened, including the isolation of patients, disinfection of the environment, practice of appropriate hand hygiene by the medical staff and disinfection of the medical instruments. In addition, when CSF gram staining shows the presence of Gram-positive bacteria, vancomycin could be used as an empirical treatment in patients who are at risk for infection with MDR bacteria. However, there are substantial challenges regarding the selection of empirical antibiotic treatments for MDR Gram-negative bacteria. If necessary, the ITH/IVT administration of colistin and aminoglycoside antibiotics could be considered.

The limitations of our study must be acknowledged. This was a single-center study with a small sample size, which may restrict the applicability of the findings to patients with CNS infections in other regions. Additionally, due to the limitations of retrospective studies, the drug sensitivity results for some antibiotics could not be obtained.

## Conclusion

It is important to know the pathogen distribution and risk factors for adverse outcomes in patients with CNS infections. Although Gram-positive bacteria are still the primary pathogens causing CNS infections, the proportion of Gram-negative organisms has increased dramatically in recent years. The detected Gram-positive bacteria were still 100% sensitive to vancomycin in our study. But, MDR/XDR *Acinetobacter baumannii* and *Klebsiella pneumoniae* accounted for high proportions. Furthermore, age > 50 years, complicated with pulmonary infections and a CSF glucose level < the normal value were independent risk factors for mortality. A CSF ADA level > the normal value and the presence of external ventricular drainage/lumbar cistern drainage were associated with MDR bacterial infections. More advanced research should be conducted on this topic.

## Data Availability

The data set supporting the conclusions in this article is available from the corresponding author on reasonable request.

## Abbreviations

ADA: Adenosine deaminase
ALP: Alkaline phosphatase
ALT: Alanine aminotransferase
AST: Aspartate aminotransferase
BBB: Blood–brain barrier
CDC: Centers for Disease Control and Prevention
CLSI: Clinical and Laboratory Standards Institute
CNS: Central nervous system
CoNS: Coagulase-negative *S**taphylococci*
CRE: Carbapenem-resistant *Enterobacteriaceae*
CSF: Cerebrospinal fluid
ESBLs: Extended-spectrum beta-lactamases
ITH: Intrathecal
IVT: Intraventricular
MDR: Multi-drug-resistant
TBIL: Total bilirubin
XDR: Extensively drug-resistant
